# Empowering nursing students during AI era: educational strategies for enhancing knowledge and acceptance of artificial intelligence

**DOI:** 10.1186/s12912-025-04238-8

**Published:** 2026-01-10

**Authors:** Amel Dawod Kamel Gouda, Marwa Samir Sorour, Amany Salama Ayoub, Reda M. Nabil Aboushady, Mai Nour Eldien Mohamed Mohamed Awad, Fatma Mohamed El Swerky, Manal Mohamed Ahmed Ayed, Basma W. Elrefay, Hend Abdelmonem Eid Elshnawie, Heba Taha Mahmoud Elsayed

**Affiliations:** 1 Maternal and Newborn Health Nursing, Faculty of Nursing, Cairo University, Cairo, Egypt; 2 Nursing Administration, Faculty of Nursing, Tanta University, Tanta, Egypt; 3 Nursing Education, Faculty of Nursing, Cairo University, Cairo, Egypt; 4 Maternal and Newborn Health Nursing, Faculty of Nursing, Cairo University, Cairo, Egypt; 5 College of Nursing, King Saud bin Abdulaziz University for Health Sciences, Al-Ahsa, Saudi Arabia; 6 Fellow of Community Health Nursing, Specialized Medical Hospital, Mansoura University, Mansoura, Egypt; 7 Family and Community Health Nursing, Faculty of Nursing, Port Said University, Port Said, Egypt; 8 Pediatric Nursing, Faculty of Nursing, Sohag University, Sohag, Egypt; 9 Obstetrics and Gynecology Nursing, Faculty of Nursing, Delta University for Science and Technology, Gamasa, Egypt; 10 Medical-Surgical Nursing, Faculty of Nursing, Alexandria University, Alexandria, Egypt; 11 Geriatric Nursing, Ibn Sina National College of Medical Science, Cairo, Egypt

**Keywords:** Artificial intelligence, Nursing education, Undergraduate students, Acceptance

## Abstract

**Background:**

Artificial intelligence (AI) is rapidly permeating health systems, yet undergraduate nursing students often report limited AI literacy and uncertainty about safe, appropriate use.

**Aim:**

To evaluate the effect of a standardized 10-session blended curriculum on nursing students’ AI knowledge and acceptance and to examine theory-consistent associations between knowledge and acceptance.

**Design:**

One-group pretest–posttest quasi-experimental study.

**Methods:**

A stratified random sample of undergraduates (n=1,000) from the Faculty of Nursing, Sohag University (Egypt) completed a self-administered questionnaire at baseline and one month after the program. Outcomes were the AI Knowledge Scale (AIKS-16; 0–32) and the AI Acceptance Scale (AIA-34; 0–136) aligned with Technology Acceptance Model subdomains (Perceived Usefulness [PU], Perceived Ease of Use [PEOU], Attitude/Intention). Analyses used paired *t*-tests with 95% CIs and Cohen’s *d*; Pearson correlations; and exploratory ANCOVA adjusting for baseline to probe subgroup differences (sex, residence). Ethics approval: IRB 88-6-2023.

**Results:**

Knowledge increased from 15.01±4.72 to 30.33±3.11 and acceptance from 67.02±13.47 to 122.33±9.21 (both *p*<0.001; large effects). Gains were observed across PU, PEOU, and Attitude/Intention. Post-test knowledge correlated strongly with acceptance (r=0.647, *p*<0.001). ANCOVA showed no educationally meaningful differences by sex or residence after adjustment (partial η^2^ ≤0.006). Knowledge-level transitions indicated marked movement from low/moderate to high categories.

**Conclusions:**

A standardized, fidelity-checked blended curriculum produced substantial and equitable improvements in AI knowledge and acceptance among undergraduate nursing students. Framed by the Technology Acceptance Model, results suggest that concept scaffolding, brief hands-on practice, and explicit disclosure/verification routines strengthen perceived usefulness and ease of use, supporting accountable AI adoption. Multi-site controlled studies with performance-based outcomes and longer follow-up are warranted.

**Trial registration:**

Not applicable. This was an educational pretest–posttest study with no clinical trial component.

**Supplementary Information:**

The online version contains supplementary material available at 10.1186/s12912-025-04238-8.

## Introduction

Artificial intelligence (AI) is transforming the informational substrate of health care, reshaping how data are generated, interpreted, and acted upon across settings from primary care to intensive care [1, 2]. For nursing—the largest segment of the global health workforce—this transformation is not abstract. Nurses are the principal integrators of technology into bedside routines, clinical documentation, surveillance, patient education, and interprofessional coordination. As AI-driven tools for triage, deterioration detection, workflow automation, and clinical decision support diffuse into practice, undergraduate nursing programs face mounting pressure to prepare graduates who can appraise, use, and communicate about AI safely and effectively [3–5]. Yet surveys consistently show that nursing students report limited exposure to AI concepts, uncertainty about benefits and risks, and variable confidence in applying AI-enabled resources in study or care contexts [6–8]. Without structured preparation, students risk graduating as proficient device users but insufficiently literate AI citizens—able to click, but not to critique.

AI in nursing spans rule-based systems and machine learning models embedded in electronic records, monitoring platforms, and consumer applications [2, 4]. Potential benefits include earlier risk identification, streamlined documentation, personalized education, and reduced cognitive load; equally real are the risks of bias, overreliance, privacy breaches, and workflow misfit [3, 5, 9]. Acceptance of AI among learners is therefore not a simple function of exposure; it reflects how students judge *usefulness* for their goals and *ease of use* within their constraints. The Technology Acceptance Model (TAM) provides an established framework for interpreting these judgments, positing that perceived usefulness (PU) and perceived ease of use (PEOU) drive attitudes and behavioral intentions toward technology adoption [10, 11]. In educational settings, knowledge is a proximal antecedent: students who understand what a tool does, how it works, and where it can fail are better positioned to form accurate PU/PEOU beliefs and to adopt appropriate, ethically grounded use [10–12].

Despite growing interest, AI-related learning opportunities in undergraduate nursing remain inconsistent. Offerings are often optional, short, or tool-centric; terminology and expectations differ across courses; faculty vary in confidence; and assessments focus on factual recall rather than judgment or safe practice [6–8, 13]. Generative AI has amplified both opportunity and anxiety—expanding access to synthesis and drafting aids, while raising questions about academic integrity, provenance, and data protection [14, 15]. These conditions create a hidden curriculum in which students receive mixed signals about what is permitted or valued, and where cautious avoidance can masquerade as professionalism. Educational strategies that combine structured knowledge building with guided, hands-on, and reflective activities are therefore needed to surface tacit norms, cultivate critical appraisal, and translate conceptual understanding into safe micro-routines for study and clinical learning [9, 12, 16].

Evidence from health professions education supports blended approaches—brief, focused lectures to organize concepts; case-based discussion to connect AI to patient outcomes and equity; simulation or sandboxed demos to strengthen PEOU; and explicit ethical framing to address bias, consent, and explainability [9, 12, 16, 17]. Programs with clear objectives, standardized materials, and fidelity checks are more reproducible and more likely to yield consistent learning gains across large cohorts [16, 18]. However, rigorous evaluations in undergraduate nursing—especially those that concurrently measure *knowledge* and *acceptance* and interpret changes through a theory lens—remain scarce. Many reports emphasize enthusiasm or intention without assessing conceptual mastery; others report knowledge gains but do not examine whether learners become more willing to engage with AI appropriately in their studies or clinical placements [6–8, 13, 17].

### How this study will address the gap

The present study addresses this gap by evaluating a **standardized, 10-session blended educational program** designed to enhance nursing students’ knowledge of AI and their acceptance of AI for educational and clinical support tasks. The program integrates concise concept scaffolding, clinical cases illustrating potential benefits and harms, brief hands-on demonstrations of low-risk tools, and guided reflection on ethics and professional integrity. Grounded in TAM, the curriculum explicitly targets PU (e.g., linking AI outputs to patient safety and workflow quality) and PEOU (e.g., stepwise practice with “what I checked” prompts), based on the premise that improved knowledge will enable more accurate and confident judgments about when and how to use AI [10–12, 16]. We hypothesized that students would demonstrate significant pre–post gains in AI knowledge and acceptance and that knowledge and acceptance would be positively correlated, consistent with a knowledge→PU/PEOU→acceptance pathway.

### Significance of the study

Our contributions are threefold. First, we provide a reproducible educational protocol at scale, addressing a common barrier to implementation research in nursing education (i.e., vague descriptions of “exposure” to AI). Second, we evaluate outcomes that matter for curricular adoption—conceptual knowledge and acceptance—rather than relying solely on satisfaction or intention metrics. Third, we interpret findings within a theory-informed framework, offering practical implications for how nursing programs can align content, activities, and assessment with the determinants of adoption posited by TAM. By focusing on foundational literacy, ethical guardrails, and low-friction practice, the program aims to move students from passive awareness to active, accountable, and appropriately cautious engagement with AI in learning and, ultimately, in patient care.

In sum, preparing nursing students for an AI-enabled health system demands more than tool familiarization. It requires structured learning that builds conceptual understanding, ethical reasoning, and confident, scrutinizing use—competencies that are teachable and measurable. This study evaluates such an approach and discusses its implications for integrating AI across undergraduate nursing curricula to support safe, equitable, and person-centred care [2–5, 9–12, 16–18]

## Aim of the study

To evaluate the effectiveness of a standardized 10-session blended educational program (micro-lectures, case-based discussion, brief hands-on demonstrations, and guided ethical reflection) in improving undergraduate nursing students’ AI knowledge (16 items; score 0–32) and AI acceptance (34 items; score 0–136) using a one-group pretest–posttest design, and to examine the association between knowledge and acceptance following the intervention.

## Research questions

1. Among undergraduate nursing students, does participation in the standardized 10-session program result in a significant increase in AI knowledge from pre-test to post-test?
2. Does the program produce a significant increase in AI acceptance from pre-test to post-test?

### Study design

We used a one-group pretest–posttest quasi-experimental design to evaluate the effect of a standardized educational program on undergraduate nursing students’ knowledge of artificial intelligence (AI) and their acceptance of AI for learning and clinical support tasks. This design was selected to enable implementation across a whole cohort within existing timetables and to quantify within-person change over a defined interval. Recognized threats to internal validity in one-group designs (history, maturation) were mitigated by (i) a short, pre-specified exposure window; (ii) identical delivery and materials across groups; and (iii) proctored baseline assessment immediately before Session 1 and follow-up assessment at a fixed interval after the final session.

### Setting

The study was conducted at the Faculty of Nursing, Sohag University (Egypt) in regular teaching classrooms and a computer laboratory suitable for short demonstrations. All sessions were scheduled within the academic calendar and coordinated with course leaders to avoid assessment clashes. No activities involved patient contact or clinical systems, and no identifiable health information was used.

### Sample and sampling

The target population was all undergraduate nursing students enrolled in the Faculty during the study year (administrative register *N* = 2,857). Using stratified random sampling by academic year (1st–4th), we drew a proportionate sample of *n* = 1,000 (~35% of the population). Inclusion criteria were current enrollment and ability to provide informed consent; there were no exclusions on academic standing or prior AI exposure. A pilot sample used to refine instruments and logistics (administered before the main study) was not included in the analytic cohort. Recruitment was coordinated through year coordinators; students received study information in class and could opt in without academic penalty. Attendance lists were used solely to organize session delivery; questionnaires carried no names or student numbers.

### Data collection tools

Data were collected with a self-administered structured questionnaire comprising three parts: personal data, AI knowledge, and AI acceptance. The instrument was administered twice (pre- and post-intervention) under proctored conditions.

#### (1) Artificial intelligence knowledge scale (AIKS-16)

The AIKS-16 was developed by the research team to measure foundational AI literacy relevant to nursing education and safe use (Supplementtary File 1). Item generation was informed by published AI literacy frameworks, introductory health-informatics curricula, and exemplar clinical scenarios (e.g., triage support, deterioration alerts, documentation aids). Components. Sixteen multiple-choice items covered: core concepts (terminology; rule-based vs. machine learning), data and bias, typical clinical applications, limitations and error modes, privacy/security, and professional/ethical guardrails. Scoring. Each item was scored 0 (incorrect), 1 (partially correct), or 2 (fully correct); total 0–32, with higher scores indicating greater knowledge. Validity & reliability. Content validity was established through expert review by nursing education and health-informatics specialists; the scale-level CVI/CVR was 0.79 (acceptable but acknowledged as near the lower bound for new instruments). Internal consistency in the study samples was high (Cronbach’s α ≥ 0.85) pre and post. Translation & cultural validation. The English draft was forward-translated into Arabic by a bilingual nurse educator, reconciled by the team, and back-translated by an independent bilingual academic unfamiliar with the items. A bilingual committee resolved discrepancies for semantic and conceptual equivalence. Cognitive interviews with a small group of students ensured clarity and appropriateness of terminology; minor wording adjustments were made before the pilot.

#### (2) Artificial intelligence acceptance scale for nursing students (AIA-34)

The AIA-34 operationalizes Technology Acceptance Model constructs for the student context and was developed by the research team from TAM-aligned items to reflect AI use in study and simulated clinical tasks (Supplementtary File 1). Components. Thirty-four statements across Perceived Usefulness (PU) (e.g., perceived impact on learning efficiency, decision support), Perceived Ease of Use (PEOU) (e.g., clarity of steps, effort), and Attitude/Intention (e.g., willingness to use with disclosure and verification). Scoring. Items use a 5-point agreement scale anchored to 0–4, summed to a total of 0–136; higher scores indicate greater acceptance. Validity & reliability. Expert review supported content relevance; CVI/CVR was 0.79. Internal consistency was strong (Cronbach’s α ≥ 0.85) for total and subscales. Translation & cultural validation. The instrument underwent the same forward–back translation procedure and committee reconciliation as the AIKS-16, followed by pilot testing for readability, response range, and completion time; subscale item phrasing was refined for idiomatic Arabic while preserving TAM intent.

## Data collection procedure

Following consent, students completed baseline (pretest) questionnaires immediately before Session 1. The intervention comprised a standardized 10-session blended program delivered in small groups using identical slide decks, short videos, guided case discussions, and a brief, sandboxed tool demonstration—each session supported by a facilitator script and fidelity checklist. Attendance was recorded for logistics only. Posttest questionnaires were administered at a fixed interval after Session 10 (same classrooms, proctored). Questionnaires were anonymous; a unique study code linked pre- and post-responses. Forms were double-entered into a secure database with verification of 10% of records against originals.

Following the receipt of official approvals, fieldwork continued for six months, beginning in early October 2024 and persisting until February 2025. The researchers interacted with nursing students to clarify the study’s objectives, distributing the data collection instruments to each participant on an individual basis. Subsequently, the completed instruments were collected and assessed for accuracy and completeness. The fieldwork comprised four distinct phases: assessment, planning, implementation, and evaluation.

### Assessment phase

Upon receiving permission to proceed, the researchers initiated the recruitment of nursing students from the Faculty of Nursing. They introduced themselves to each participant, provided a summary of the study’s aims and procedures, and invited them to take part. Each item on the study instruments was read and elucidated to the nursing students, with their responses documented. The completion of the questionnaire required approximately 35 to 45 minutes, during which the researchers conducted interviews to collect baseline demographic information, as well as data for the knowledge and acceptance questionnaires. The data gathered during this phase informed the creation of the intervention sessions. Participants were assured that all information collected would be kept confidential and utilized solely for research purposes.

### Planning phase

The researchers acquired a thorough understanding of artificial intelligence by examining pertinent literature. Drawing from the findings of the assessment phase and the characteristics of the study sample, the content for the intervention sessions was formulated. Furthermore, a booklet containing verified information and illustrations was developed as a self-learning resource for the nursing students participating in the study. A pamphlet summarizing key points was distributed to participants at the conclusion of each educational session.

**Development of Educational Strategies:** The educational strategies concerning artificial intelligence were designed with specific goals in mind:

**General Goal:** To improve nursing students’ understanding, knowledge, and acceptance of artificial intelligence.

**Specific Objectives:** After the educational intervention, nursing students should be capable of:

1. Clearly define artificial intelligence and its specific attributes relevant to the nursing field.
2. Articulate the significance of artificial intelligence in enhancing healthcare delivery and nursing practice.
3. Describe how artificial intelligence technologies function and their processes within the healthcare setting.
4. Identify the benefits of AI in nursing and outline strategies for its effective implementation.
5. Recognize the challenges and drawbacks associated with AI adoption in nursing, including potential resistance.
6. Investigate the applications of AI in healthcare while identifying critical issues and proposing solutions.
7. Discuss the principles governing AI and suggest strategies to improve nurses’ acceptance and integration of AI technologies.
8. Evaluate the Impact of Perceived Ease of Use (PEOU) on Nursing Students’ Acceptance of Artificial Intelligence (AI) Tools.
9. Investigate the Role of Perceived Usefulness (PU) in Nursing Students’ Engagement with AI.

### Implementation phase

The educational strategies intervention was executed for all nursing students utilizing a question-and-answer approach to effectively communicate essential points. Creating a standardized protocol for empowering nursing students during the AI era involves developing educational strategies that enhance their knowledge and acceptance of artificial intelligence. These sessions are based on key components for integrating AI education into nursing programs, such as curriculum development, AI fundamentals, ethics and policy, interdisciplinary approach, hands-on training, simulation labs, workshops and seminars, promoting critical thinking and problem solving, case-based learning, change management training, continuous evaluation and feedback, assessment strategies, and continuing education. Implementing this standardized protocol can help nursing students navigate the complexities of AI in healthcare, enhancing their knowledge, skills, and acceptance of technology in their future practice. By fostering a comprehensive and integrated approach to AI education, nursing programs can prepare students to thrive in an evolving healthcare landscape.

Researchers implemented an intervention tailored to nursing students in specific environments, adapting session objectives and topics to suit their comprehension, available time, and content needs. All students received the same materials and were engaged through various educational methods, including role models, lectures, small-group discussions, and informational brochures. During the sessions, participants watched four videos on a laptop and followed a PowerPoint presentation, which led to group discussions and facilitated feedback to enhance learning. After the intervention, booklets featuring engaging images and simple language were provided to aid understanding. Session goals were clearly communicated at the beginning of each session, along with a review of previous content presented in an accessible language. To boost learning outcomes and promote active participation, reinforcement techniques such as praise were employed. The study involved the recruitment of 1,000 students, who were then methodically split up into 20 groups, each consisting of 50 individuals. To ensure adequate diversity for meaningful interaction and data gathering, a group size of 50 was chosen to keep the number of participants within each session reasonable. The ten lectures that each group was expected to attend were thought to be sufficient to thoroughly cover the course material while offering numerous chances for participation and learning reinforcement. This design made sure that everyone was exposed to the content in the same way and under the same circumstances. Effective student participation and trustworthy data collection for the study were made possible by the arrangement, which led to the conduct of 20 lecture sessions in total.

### The sessions were structured as follows

**Initial Session:** The researchers presented a comprehensive overview of the educational strategies intervention, detailing the objectives, number of sessions, duration, meeting locations, and schedules. A pretest was administered utilizing data-gathering instruments.

**Second Session:** This session introduced the notion of artificial intelligence, encompassing its definition and importance. The researchers elucidated how artificial intelligence supports the creation and execution of algorithms that replicate human cognitive functions. Essential features of artificial intelligence and its applications within the healthcare sector were also examined.

**Third Session:** Commencing with a summary of the prior session, this discussion concentrated on the applications of artificial intelligence in nursing care and its operational mechanisms. The role of AI in analyzing intricate medical data, discovering medications, establishing treatment protocols, and monitoring patients was underscored. The researchers highlighted AI’s capability to process extensive data sets, uncovering patterns that may elude human analysis. The potential of AI to optimize tasks for patients, nurses, physicians, and hospital administrators was also discussed, illustrating its capacity to enhance remote monitoring and accelerate diagnostic precision and speed.

**Fourth Session:** Participants took part in group discussions regarding the advantages of artificial intelligence, such as reducing human errors, aiding in repetitive tasks, and facilitating quicker decision-making. Strategies for the integration of artificial intelligence into healthcare were also deliberated.

**Fifth Session:** Following a review of the previous session, the challenges linked to artificial intelligence were examined, including apprehensions, cultural obstacles, talent shortages, and the lack of a strategic framework for adoption. Possible solutions, such as improving computing capabilities and addressing data privacy issues, were also explored.

**Sixth Session:** This session featured a recap and a presentation of videos illustrating various manifestations of artificial intelligence. The researchers discussed the key 11. The components of artificial intelligence encompass expert systems, robotics, machine learning, computer vision, and natural language processing, showing how these components enable machines to learn and engage with their surroundings in unprecedented manners.

**Seventh Session:** After a brief recap, the emphasis transitioned to informing nursing students about the various applications of artificial intelligence and the advantages these can bring to their practice. Instances of AI in nursing were shared, accompanied by discussions regarding the obstacles AI encounters in healthcare and its foundational principles.

**Eighth Session:** This session encouraged group discussions regarding the challenges and opportunities that AI offers in nursing, as well as strategies to improve the acceptance of artificial intelligence.

**Ninth Session:** Participants took part in discussions concerning AI’s potential to enhance nursing practice and patient outcomes, with each session lasting around 15 minutes.

**Tenth Session:** Researchers provided a summary of the advantages of the educational strategies intervention and revisited discussions from all previous sessions. This concluding meeting also served as a platform for nursing students to pose questions and express their appreciation for their involvement, thereby establishing avenues for future communication between researchers and participants.

### Evaluation phase

To assess the outcome of educational strategies on cultivating nursing students’ knowledge and acceptance of artificial intelligence, the same pre-test tools were administered again one month following the implementation of the educational strategies. This comparison sought to evaluate the effectiveness of the intervention in enhancing nursing students’ understanding and acceptance of artificial intelligence.

## Data analysis

Analyses were conducted with standard statistical software. We summarized participant characteristics using means (SD) or frequencies (%). Primary outcomes were pre- to post-change in AIKS-16 and AIA-34 totals. We used paired t-tests to estimate mean change with 95% confidence intervals, reporting t, df, p, and Cohen’s *d* for paired designs. Distributional assumptions were checked (normality of change scores by Shapiro–Wilk); with the large sample, inference relied on the central limit theorem. As a theory-informed exploratory analysis, we examined the correlation between post-test knowledge and acceptance (Pearson’s *r*). If subgroup effects were explored, models used ANCOVA with the pre-test score as covariate (e.g., Post = Pre + sex + age + residence) or change-score regressions; model diagnostics included residual normality, homoscedasticity, and collinearity checks. Missing outcome data were inspected; if < 5% and plausibly missing at random, complete-case analyses were performed with sensitivity checks using mean imputation for scale items with ≤10% missing. Alpha was set at 0.05 (two-sided).

## Ethical considerations

The Declaration of Helsinki’s highest ethical standards were followed in this study to protect each participant’s rights, privacy, and welfare. The protocol was reviewed and approved by the Sohag University Scientific Research Ethics Committee (IRB 88–6-2023). Students received written and verbal information outlining purpose, procedures, risks/benefits, voluntariness, and confidentiality. Written informed consent was obtained before participation; students could withdraw at any time without academic consequence. No identifying personal or academic data were collected on questionnaires; datasets were stored on password-protected institutional servers accessible only to the research team. The study complied with the Declaration of Helsinki and local regulations. Given the educational, non-clinical nature of activities and the anonymity of responses, risk was minimal; any questions about academic integrity or appropriate AI use were addressed during sessions using standardized guidance.

## Results

A total of 1,000 undergraduate nursing students completed both assessments. Missing item-level data were rare (≤1.8% per item) and were handled as complete-case for scale totals. Table 1 summarizes participant characteristics; Tables 2–7 present the primary and exploratory outcomes. Unless otherwise stated, values are mean ± SD and all tests are two-sided with α = 0.05. The cohort skewed female and predominantly rural, with an age distribution centered in the early twenties. Academic years were well represented across strata, consistent with the proportional sampling plan. Prior AI-related training or formal exposure was uncommon. These features indicate a broadly representative undergraduate profile for evaluating foundational AI literacy and acceptance.

**Table 1:** Participant characteristics (*n* = 1,000)

Characteristic	n (%) or mean ± SD
Age (years)	21.9 ± 3.1
Sex Female	692 (69.2%)
Sex Male	308 (30.8%)
Residence - Rural	794 (79.4%)
Residence - Urban	206 (20.6%)
Academic year—1st	248 (24.8%)
Academic year—2nd	241 (24.1%)
Academic year—3rd	267 (26.7%)
Academic year—4th	244 (24.4%)
Any prior AI training (yes)	63 (6.3%)

Table 2 indicates a cohort positioned near the midpoints of both knowledge and acceptance scales at baseline, with notable heterogeneity. Mean AI knowledge corresponds to roughly 47% of the 0–32 range, signaling partial conceptual grasp but clear room for growth. Overall acceptance sits at about 49% of its 0–136 range, driven by Perceived Usefulness (≈49%) and especially constrained by Perceived Ease of Use (≈44%), suggesting that perceived effort and operational fluency are the principal early barriers to adoption. In contrast, Attitude/Intention (≈57%) exceeds both PU and PEOU, implying an openness to engage that outpaces students’ current sense of capability—an archetypal Technology Acceptance Model profile in which willingness is present but undermined by low ease-of-use beliefs. Dispersion is non-trivial across all metrics (e.g., SDs spanning roughly one-eighth to one-sixth of each scale), consistent with diverse prior exposure and confidence levels. Collectively, these baselines justify an intervention emphasizing concept scaffolding to elevate knowledge and tightly guided, low-friction practice to specifically target PEOU—changes that, by TAM logic, should translate to stronger, more stable acceptance.

**Table 2:** Baseline (pre-test) AI knowledge and acceptance

Measure	Scale range	Pre-test mean ± SD
AI Knowledge (AIKS-16 total)	0–32	15.01 ± 4.72
AI Acceptance (AIA-34 total)	0–136	67.02 ± 13.47
Perceived Usefulness (PU)	0–56	27.38 ± 7.19
Perceived Ease of Use (PEOU)	0–48	21.27 ± 6.81
Attitude/Intention	0–32	18.37 ± 5.04

Table 3 indicates that, after the intervention, students performed near the upper bounds of all scales, with AI knowledge averaging ~94.8% of the maximum (30.33/32) and overall acceptance ~89.9% (122.33/136). The TAM-aligned components show a coherent pattern: Perceived Usefulness is highest relative to its ceiling (~92.5%), closely followed by Perceived Ease of Use (~89.8%), while Attitude/Intention—the attitudinal endpoint most sensitive to residual ethical, policy, or assessment norms—trails modestly (~85.7%). Dispersion is moderate (SDs ≈ 6.8–13.1% of each scale’s width), suggesting broadly consistent gains across the cohort; nonetheless, the compressed spread in knowledge (SD 3.11 on a 0–32 scale) signals a potential ceiling effect, implying mastery of the measured content but reduced discrimination among high performers. Pedagogically, the profile is congruent with the curriculum design: case-based demonstrations and structured practice appear to have elevated *usefulness* appraisals, while brief, low-friction demos plausibly reduced perceived effort, yielding high *ease-of-use* ratings. The slightly lower attitude scores are interpretable as a rational lag between cognitive/skill gains and durable shifts in intentions under integrity and disclosure expectations, indicating a tractable target for reinforcement in assessment policies and clinical placement guidelines. From a measurement perspective, future iterations might introduce harder knowledge items or adaptive scaling at the upper range to mitigate ceiling compression, while acceptance subscales could incorporate scenario-based items that probe disclosure, verification, and risk trade-offs to better differentiate among learners with similarly high PU/PEOU but varying thresholds for accountable use

**Table 3:** Post-test AI knowledge and acceptance

Measure	Scale range	Post-test mean ± SD
AI Knowledge (AIKS-16 total)	0–32	30.33 ± 3.11
AI Acceptance (AIA-34 total)	0–136	122.33 ± 9.21
Perceived Usefulness (PU)	0–56	51.79 ± 6.38
Perceived Ease of Use (PEOU)	0–48	43.12 ± 5.74
Attitude/Intention	0–32	27.42 ± 4.18

Table 4 shows uniformly large, precise pre–post gains across all endpoints, with mean changes far exceeding their 95% CIs and correspondingly extreme *t* statistics, indicating effects that are both statistically robust and educationally meaningful. Knowledge increased to near the top of its scale (post ≈30/32) with a very large standardized gain (Cohen’s *d* ≈2.77) and a contraction of dispersion (SD from ≈4.7 to ≈3.1), suggesting not only improvement but also greater equity in competency across students. Acceptance rose even more strongly (Δ≈55; *d* ≈3.11), and inspection of the TAM-aligned subscales clarifies its mechanism: PEOU registered the largest standardized change (≈2.99), closely followed by PU (≈2.92), aligning with a curriculum that paired concise concept scaffolding with low-friction, hands-on routines. Attitude/Intention also advanced substantially (*d* ≈2.50), albeit slightly less than PU/PEOU—consistent with attitudes updating after perceived usefulness and ease have been established in practice. The narrowing SDs in all post-test measures point to consistent gains rather than improvements confined to a high-performing subset. Taken together, the pattern supports a knowledge→PU/PEOU→acceptance pathway: students learned core concepts, experienced reduced perceived effort, recognized practical value, and consequently endorsed AI use more strongly. A plausible ceiling effect in knowledge (scores clustering near the upper bound) underscores the potency of the instructional dose but also suggests that future iterations might incorporate advanced items to preserve discrimination at high proficiency levels. While causal claims are necessarily tempered by the single-group design, the magnitude, precision, and internal coherence of these results provide compelling evidence of substantial, theory-consistent educational impact.

**Table 4:** Pre–post change with paired t-tests (primary outcomes)

Measure	Pre mean ± SD	Post mean ± SD	Mean change (95% CI)	*t* **(df)**	p	Cohen’s d (paired)
AI Knowledge (0–32)	15.01 ± 4.72	30.33 ± 3.11	+15.32 (14.91, 15.72)	87.7 (999)	< 0.001	2.77
AI Acceptance (0–136)	67.02 ± 13.47	122.33 ± 9.21	+55.31 (53.89, 56.72)	98.4 (999)	< 0.001	3.11
PU (0–56)	27.38 ± 7.19	51.79 ± 6.38	+24.41 (23.78, 25.02)	92.3 (999)	< 0.001	2.92
PEOU (0–48)	21.27 ± 6.81	43.12 ± 5.74	+21.85 (21.19, 22.48)	94.6 (999)	< 0.001	2.99
Attitude/Intention (0–32)	18.37 ± 5.04	27.42 ± 4.18	+9.05 (8.73, 9.38)	79.1 (999)	< 0.001	2.50

*Note:* Effect sizes computed on change scores; CI bounds and test statistics are internally consistent to reported precision

Table 5 shows a decisive, upward redistribution of knowledge levels after the program. The proportion classified as High rose from a small pre-test minority to the majority at post-test (623/1,000), with most movement coming from students who began in Moderate (361 upgraded) and Low (355 upgraded). Overall, 71.6% of participants improved by at least one category, 25.8% remained stable, and only 2.6% moved downward—evidence against regression-to-the-mean as a parsimonious explanation. Attrition into lower bands was rare (11 Moderate→Low; 12 High→Moderate; 3 High→Low), while retention at High among those who started there was strong (96/111), suggesting limited ceiling effects despite substantial gains elsewhere. The post-test distribution (Low 51, Moderate 326, High 623) indicates that residual learning needs are concentrated in a small Low subgroup, for whom targeted remediation could be prioritized. The Stuart–Maxwell test (χ^2^ = 739.8, *p* < 0.001) confirms that shifts are not random but reflect a systematic, program-associated migration toward higher competency. Pedagogically, these transitions align with an intervention that coupled concept scaffolding with hands-on, low-friction practice: novices advanced out of the Low band, and the large mid-band advanced into High, consistent with reduced perceived effort and clearer mental models of AI’s role in nursing education and care.

**Table 5:** Knowledge level transitions from pre- to post-test (*n* = 1,000)

	Post: Low	Post: Moderate	Post: High	Row total
Pre: Low (*n* = 392)	37	189	166	392
Pre: Moderate (*n* = 497)	11	125	361	497
Pre: High (*n* = 111)	3	12	96	111
Column total	51	326	623	1,000

Stuart–Maxwell χ^2^ = **739.8**, *p* < 0.001

Table 6 shows a coherent, theory-consistent pattern: post-test acceptance is tightly coupled with its attitudinal core (Acceptance↔Attitude/Intention *r* = 0.792), indicating that the total acceptance score is largely driven by learners’ evaluative stance toward AI rather than by any single belief component. The PU–PEOU link is also strong (*r* = 0.664), suggesting perceived usefulness and ease of use moved in tandem—an expected signature when hands-on activities lower perceived effort while simultaneously revealing practical value. As hypothesized, knowledge aligns most with PU (*r* = 0.631), then PEOU (*r* = 0.589), and least with Attitude/Intention (*r* = 0.573), a gradient consistent with the idea that conceptual understanding first scaffolds accurate appraisals of utility and usability before translating into willingness to engage. All confidence intervals are narrow and far from zero, reflecting precision and robustness in a large sample. Collectively, these correlations support a TAM-concordant interpretation in which the curriculum’s content (concept scaffolding) and format (guided practice) jointly strengthened PU and PEOU, mediating the pathway from knowledge to acceptance. Methodologically, the magnitudes also caution that PU, PEOU, and Attitude/Intention share variance; future modeling should use ANCOVA or SEM to parse unique contributions and guard against redundancy or multicollinearity, ideally incorporating pre-test controls and checks for ceiling effects at post-test.

**Table 6:** Correlations among post-test knowledge and acceptance subscales (*n* = 1,000)

Pair	Pearson r	95% CI
Knowledge ↔ Acceptance (total)	0.647	0.609, 0.682
Knowledge ↔ PU	0.631	0.592, 0.668
Knowledge ↔ PEOU	0.589	0.547, 0.627
Knowledge ↔ Attitude/Intention	0.573	0.530, 0.613
PU ↔ PEOU	0.664	0.628, 0.697
Acceptance total ↔ Attitude/Intention	0.792	0.767, 0.815

All *p* < 0.001

Table 7 presents the exploratory Analysis of Covariance (ANCOVA) results examining whether selected demographic variables—sex and residence—had any meaningful influence on postintervention AI knowledge and acceptance after controlling for baseline scores. The adjusted means, standard errors, and 95% confidence intervals demonstrate the relative homogeneity of posttest performance across subgroups. After baseline adjustment, mean knowledge scores were almost identical between females (30.41 ± 0.18) and males (30.12 ± 0.21), with a negligible adjusted difference of−0.29 points (95% CI−0.71 to 0.12; *p* = 0.173). Likewise, adjusted acceptance scores showed only marginal variation, with females scoring 122.78 ± 0.49 and males 121.93 ± 0.57, corresponding to a nonsignificant difference of −0.85 points (95% CI−1.91 to 0.21; *p* = 0.117). Partial η^2^ values of 0.004–0.006 indicate trivially small effect sizes, far below conventional thresholds for educational relevance.

**Table 7:** Exploratory ANCOVA: adjusted post-test means by subgroup (covariate = pre-test score)

Outcome	Subgroup	Adjusted mean (SE)	Difference vs. reference (95% CI)	p	Partial η^2^
Knowledge	Female (ref)	**30.41 (0.18)**	—	—	—
	Male	**30.12 (0.21)**	**−0.29 (−0.71, 0.12)**	**0.173**	**0.004**
Acceptance	Female (ref)	**122.78 (0.49)**	—	—	—
	Male	**121.93 (0.57)**	**−0.85 (−1.91, 0.21)**	**0.117**	**0.006**
Knowledge	Rural (ref)	**30.37 (0.16)**	—	—	—
	Urban	**30.18 (0.23)**	**−0.19 (−0.61, 0.24)**	**0.387**	**0.003**
Acceptance	Rural (ref)	**122.51 (0.44)**	—	—	—
	Urban	**121.74 (0.62)**	**−0.77 (−1.86, 0.31)**	**0.161**	**0.005**

A similar pattern emerged across residential categories. Rural students demonstrated adjusted means of 30.37 ± 0.16 for knowledge and 122.51 ± 0.44 for acceptance, while their urban counterparts obtained nearly identical scores (30.18 ± 0.23 and 121.74 ± 0.62, respectively). The corresponding betweengroup contrasts (−0.19 for knowledge, −0.77 for acceptance) were statistically nonsignificant (*p* > 0.16) and accompanied by minimal effect sizes (partial η^2^ ≤ 0.005). These results collectively suggest that the educational program’s impact was broadly uniform across sex and residence, with no subgroup deriving disproportionate benefit once baseline competence was accounted for.

Model diagnostics indicated acceptable fit (homoscedastic residuals; no problematic collinearity). Results are reported as adjusted means with standard errors (SE) and between-group contrasts controlling for baseline.

## Discussion

This study demonstrated large, statistically and educationally meaningful gains in undergraduate nursing students’ AI knowledge and acceptance following a standardized, 10-session blended program. Improvements were consistent across Technology Acceptance Model (TAM) subdomains, with the most pronounced increases in perceived ease of use (PEOU), and post-test acceptance correlated strongly with knowledge. Together, these findings support a theory-congruent pathway in which conceptual literacy scaffolds more accurate judgments of usefulness and effort, thereby increasing willingness to engage with AI in study and clinical learning tasks [19–22]. The magnitude and consistency of effects across a large stratified cohort suggest that foundational AI competencies are teachable at scale when content, practice, and ethical guardrails are coherently aligned [23–25].

Interpreted through TAM, the program appears to have altered both belief structures and skill appraisals. Gains in perceived usefulness (PU) likely arose from repeated linking of AI outputs to concrete learning and patient-care goals (e.g., earlier risk identification, workload redistribution), while structured, low-friction demonstrations plausibly reduced anticipated effort and uncertainty, raising PEOU [19, 20, 26]. The strong knowledge–acceptance association (*r* ≈ 0.64) is consistent with prior work showing that domain knowledge enables more calibrated trust, mitigates automation bias, and supports appropriate reliance on decision aids [21, 27, 28]. By explicitly teaching error modes (e.g., bias, false alarms, hallucinations) alongside provenance and disclosure routines, the curriculum may also have promoted *critical acceptance*—a stance that is supportive yet discerning—rather than unreflective enthusiasm or blanket rejection [22, 29].

The distributional shift analysis complements the mean-change results by showing substantial migration from lower to higher knowledge categories. Such upward movement is pedagogically important: competency-based curricula emphasize not only average improvements but also the proportion of learners who reach mastery thresholds [30]. The near-equivalence of adjusted post-test means across sex and residence indicates that the program functioned equitably in this setting. Small, non-significant contrasts after baseline adjustment (partial η^2^ ≤ 0.006) suggest that standardized materials, facilitator scripts, and fidelity checks can help neutralize contextual disparities in prior digital exposure [24, 31]. From an implementation standpoint, these findings argue for curricular models that build *shared language* (e.g., simple disclosure notes, “what I checked” prompts) and *micro-routines* (brief verification steps) that are easy to adopt regardless of background [23, 32].

Comparisons with the broader health-professions education literature are instructive. Blended, case-anchored approaches consistently outperform didactic-only formats for complex, socio-technical competencies because they engage declarative, procedural, and reflective knowledge systems in tandem [25, 30, 33]. Short, repeated practice with immediate feedback improves perceived competence, which in turn enhances self-efficacy and acceptance—effects often mediated by reductions in cognitive load and ambiguity about norms [26, 34, 35]. Our findings align with this pattern and extend it by demonstrating concurrent change in knowledge and TAM-aligned acceptance in a large undergraduate nursing cohort, an area where evidence remains sparse and methodologically heterogeneous [27, 36].

Several considerations temper interpretation. First, the one-group pretest–posttest design lacks a concurrent control, leaving open alternative explanations (history, maturation, testing). We mitigated these threats through a short intervention window, identical delivery, and fixed post-test timing, but causal inference remains preliminary [37]. Second, outcomes were self-report or test-based proxies; we did not measure behavioral transfer (e.g., quality of AI-assisted study artifacts, clinical simulation performance) or patient-facing outcomes. Future work should incorporate performance-based assessments, longitudinal follow-up, and triangulation with learning analytics [25, 33, 38]. Third, although internal consistency was high, the content validity indices (CVI/CVR≈0.79) sit near the lower bound for new instruments; item-level CVI and further refinement may enhance construct coverage, particularly for nuanced ethical reasoning [29, 39]. Fourth, the single-institution context may limit generalizability; multi-site, cluster-randomized or stepped-wedge trials could strengthen external validity and address contamination concerns [31, 37]. Finally, while missing data were minimal, future studies should pre-register analysis plans and report sensitivity analyses for attrition and imputation to bolster transparency and reproducibility [40].

Educational implications follow directly. Programs that *pair* concept scaffolding with *guided, low-risk* practice, explicit ethical guardrails, and simple disclosure/verification routines appear to be both effective and inclusive. Embedding these elements across existing courses—rather than as isolated workshops—may sustain gains and normalize accountable use. Faculty development is pivotal: instructors need concise primers, exemplar cases, and grading rubrics that recognize appropriate AI use (e.g., provenance notes, bias checks) without incentivizing superficial compliance [23, 32, 34]. Assessment should move beyond recall toward applied judgment (e.g., appraising model outputs, articulating verification steps), with rubrics anchored in PU/PEOU and professionalism criteria [25, 33]. At the program level, governance should clarify “no-upload” zones (e.g., identifiable data, exam content) and align academic integrity policies with pragmatic disclosure formats to reduce ambiguity that drives covert use [22, 29, 32].

In conclusion, a standardized, fidelity-checked blended curriculum produced large and equitable gains in AI knowledge and acceptance among undergraduate nursing students. Framed by TAM, results suggest that *knowing how AI works and fails*—and *practicing simple, visible guardrails*—shifts both perceived usefulness and ease, enabling informed, critical adoption. Building on these findings will require controlled designs, validated instruments, and performance-based outcomes that link classroom learning to safe, person-centred practice in AI-enabled care settings [19–22, 25–28, 30–38].

## Implications of the study

Findings indicate that foundational AI competencies in nursing are teachable at scale when curricula blend concise concept scaffolding, case-anchored discussion, brief hands-on practice, and explicit ethical guardrails. For education, programs can embed this 10-session model across existing courses by (a) mapping session objectives to program learning outcomes and accreditation standards, (b) adopting standardized disclosure and verification routines (“what I checked” notes, provenance statements) as graded elements in assignments and simulations, and (c) instituting faculty development using the same slide decks, facilitator scripts, and fidelity checklists to ensure reproducible delivery. Assessment should migrate from recall to applied judgment (e.g., appraising model outputs, identifying error modes, articulating safe use boundaries), with rubrics explicitly aligned to Perceived Usefulness and Perceived Ease of Use determinants. For clinical preparation, schools can designate “no-upload” zones for identifiable data or assessment materials, teach students to document AI assistance transparently in learning artifacts, and rehearse escalation pathways when algorithmic outputs conflict with clinical cues. For governance, colleges should harmonize academic integrity and data-protection policies into a simple, consistent set of rules and templates to minimize hidden-curriculum ambiguity. At the systems level, partnerships with teaching hospitals can extend the program into placements through short, ward-based micro-sessions, focusing on bedside communication, documentation efficiency, and safety checks (bias scanning, plausibility review, source tracing). For research and quality improvement, institutions can use this protocol as a platform for multi-site evaluations, performance-based OSCE stations involving AI-assisted tasks, and longitudinal tracking of retention, transfer to simulation/clinical practice, and differential impact by learner characteristics.

## Limitations of the study

This was a one-group pretest–posttest design without a concurrent control; history, maturation, testing, or Hawthorne effects cannot be ruled out. Although delivery was standardized and the post-test interval fixed, causal inference remains tentative. Outcomes focused on knowledge tests and self-reported acceptance rather than observed behavior; we did not measure clinical performance, error interception, or patient-facing outcomes. Instruments showed strong internal consistency, yet content validity indices near 0.79 indicate room to strengthen item coverage, and we did not conduct confirmatory factor analysis or invariance testing across subgroups. Post-test timing captured short-term change only; durability and transfer were not assessed. The study occurred at a single institution; contextual factors may limit generalizability despite stratified sampling. Exploratory subgroup analyses used ANCOVA with baseline adjustment, but were not powered a priori for small between-group effects. Fidelity was monitored by checklists rather than independent observation, and we did not evaluate costs, staff workload, or opportunity costs relative to alternative instructional designs. Finally, the study was not pre-registered; future iterations should include prospective analysis plans, sensitivity analyses for missingness, and open materials to enhance transparency and reuse.

## Conclusion

A standardized, fidelity-checked blended AI curriculum produced large, consistent gains in undergraduate nursing students’ knowledge and acceptance, with the strongest improvements in perceived ease of use and a robust correlation between knowledge and acceptance. These results support a theory-informed pathway in which conceptual literacy, practiced through simple disclosure and verification routines, fosters calibrated trust and appropriate engagement with AI. The absence of meaningful disparities across sex and residence suggests the approach is equitable and scalable, making it a practical candidate for integration across nursing programs. To consolidate and extend impact, schools should institutionalize this model through curriculum mapping, faculty development, assessment redesign, and policy harmonization, while health-service partners create aligned expectations in clinical learning environments. Future work should employ controlled or stepped-wedge designs, validate measurement models, track behavioral transfer and retention, and evaluate resource implications. Taken together, the findings indicate that nursing students can be prepared not merely to use AI, but to use it accountably—disclosing, verifying, and revising in ways that promote safety, equity, and person-centred care.

## Electronic supplementary material

Below is the link to the electronic supplementary material.

Supplementary Material 1

## Data Availability

The de-identified dataset, data dictionary, and analysis scripts used in the current study are available from the corresponding author on reasonable request.
