# Supplementary material for: Empowering nursing students during AI era: educational strategies for enhancing knowledge and acceptance of artificial intelligence
Source: BMC Nurs. 2026 Jan 10;25:95. doi: 10.1186/s12912-025-04238-8 (PMC12849734; doi:10.1186/s12912-025-04238-8)
Supplement: Supplementary file 1 — Supplementary Material 1 [file 12912_2025_4238_MOESM1_ESM.docx]

**Structured Interview Regarding Nursing Students' Knowledge and Acceptability of AI**

**Part I:** **Personal data of nursing students**

**Age:** **1- < 20 2- > 20**

**Gender: 1- Male 2-Female**

**Residence :** **1- Rural 2- Urban**

**Part II: Structured Interview Regarding Nursing Students' Knowledge of AI**

**Structured Interview Regarding Nursing Students' Knowledge of AI**

**1. Definition of AI (Questions 1-3)**

1. What is your understanding of Artificial Intelligence (AI)? Can you provide a brief definition?

- (Follow-up: How does this definition relate to the healthcare field?)

2. What are some of the key characteristics that define AI technologies?

- (Follow-up: In your opinion, how do these characteristics apply to nursing practice?)

3. Can you differentiate between narrow AI and general AI? Provide examples relevant to healthcare.

- (Follow-up: How do you think these types of AI impact patient care?)

**2. Significance of AI (Questions 4-6)**

4. Why do you think AI is important in the field of nursing?

- (Follow-up: Can you name any specific areas in nursing where AI can make a significant impact?)

5. What role do you believe AI can play in improving patient outcomes?

- (Follow-up: Can you provide any examples or scenarios where this might be applicable?)

6. How does AI integration in healthcare influence the role of nurses?

- (Follow-up: What skills do you think nurses need to develop to work effectively with AI?)

**3. Operational Mechanisms (Questions 7-9)**

7. Can you explain how AI systems learn and make decisions?

- (Follow-up: What is machine learning, and how is it relevant to healthcare?)

8. What is the role of data in the functioning of AI applications in nursing?

- (Follow-up: How do you think data integrity affects AI outcomes in healthcare?)

9. Can you describe any AI tools or systems that are currently in use in nursing practice?

- (Follow-up: What features do these tools have that assist in nursing tasks?)

**4. Various Types of AI (Questions 10-12)**

10. What are some different types of AI technologies you are aware of (e.g., natural language processing, robotics, etc.)?

- (Follow-up: Which types do you think are most relevant to your future nursing career?)

11. How do you think predictive analytics in AI can benefit nursing professionals?

- (Follow-up: Can you give examples of diseases or conditions that might benefit from predictive analytics?)

12. What is your understanding of chatbots and virtual assistants in healthcare? How do you see them being used in nursing?

- (Follow-up: Are there any potential risks associated with these technologies?)

**5. Advantages of AI (Questions 13-14)**

13. What do you believe are the primary advantages of implementing AI in nursing?

- (Follow-up: Can you discuss any potential improvements in efficiency or accuracy?)

14. How do you think AI can alleviate some common problems faced in nursing, such as staffing shortages or administrative burdens?

- (Follow-up: Can you suggest any specific AI solutions that could help in these areas?)

**6. Disadvantages of AI (Questions 15-16)**

15. What concerns do you have about the use of AI in nursing?

- (Follow-up: Can you think of any ethical dilemmas that may arise from AI applications?)

16. In your opinion, what are the potential risks or disadvantages of relying on AI in patient care?

- (Follow-up: How can nursing professionals mitigate these risks while using AI?)

**Part III: Structured Interview Regarding Nursing Acceptability of AI**

**Structured Interview Items on Nursing Acceptability of AI**

**General Perceptions of AI**

1. I believe that AI can enhance the quality of patient care in nursing.

2. I am comfortable using AI tools in clinical settings.

3. AI technologies can improve the efficiency of nursing tasks.

4. I find AI intimidating when it comes to patient interaction.

**Knowledge and Training**

5. I feel adequately trained to use AI technologies in nursing.

6. I would like to receive more education about AI applications in healthcare.

7. I am confident in my ability to adapt to new AI technologies.

8. Knowledge of AI is essential for modern nursing practice.

**Impact on Patient Care**

9. AI can help in making more accurate diagnoses.

10. AI applications can lead to better patient monitoring and follow-up.

11. I believe that AI can assist nurses in making clinical decisions.

12. AI tools may replace the need for human judgment in patient care.

**Ethical Considerations**

13. I have concerns about the ethical implications of using AI in nursing.

14. I believe that using AI raises issues related to patient privacy.

15. AI systems should be transparent in their decision-making processes.

16. The use of AI may lead to depersonalization of patient care.

**Job Security and Professional Roles**

17. I worry that AI might replace nursing jobs in the future.

18. I believe that AI will enhance the role of nurses rather than replace it.

19. AI can help me focus on more critical aspects of patient care.

20. The integration of AI in nursing practice will lead to a devaluation of the nursing profession.

**Collaboration and Teamwork**

21. I think AI can facilitate better teamwork among healthcare professionals.

22. AI will help create a collaborative environment in healthcare settings.

23. I feel that AI can assist in communication with other healthcare providers.

24. The role of AI should be limited to technical support, not decision-making.

**Innovation and Advancement**

25. I am excited about the potential innovations AI could bring to nursing.

26. AI technologies should be regularly updated and improved in healthcare.

27. I believe AI has the potential to revolutionize nursing practices.

28. Innovations in AI should prioritize patient safety and care quality.

**Acceptance and Trust**

29. I trust AI technologies to operate effectively in healthcare.

30. I believe that patients will accept AI tools if they improve care quality.

31. I would personally recommend AI tools to my peers in nursing.

32. I feel that the benefits of AI outweigh the potential risks.

**Future Outlook**

33. I see the future of nursing being closely linked with AI advancements.

34. I believe that understanding AI is crucial for my career development in nursing.
